# Serum lipoprotein(a) and risk of contrast-induced nephropathy in patients with type 2 diabetes mellitus

**DOI:** 10.3389/fcvm.2026.1733119

**Published:** 2026-02-27

**Authors:** Yesheng Ling, Yang Chen, Xianguan Yu, Ximei Zhang

**Affiliations:** Department of Cardiology, The Third Affiliated Hospital, Sun Yat-Sen University, Guangzhou, Guangdong, China

**Keywords:** contrast-induced nephropathy, coronary angiography, percutaneouscoronary intervention, serum lipoprotein(a), type 2 diabetes mellitus

## Abstract

**Aim:**

To assess the predictive value of serum lipoprotein(a) [Lp(a)] for contrast-induced nephropathy in patients with type 2 diabetes mellitus (T2DM).

**Methods:**

Consecutive T2DM patients who underwent coronary angiography (CAG) or percutaneous coronary intervention (PCI) between January 2019 and December 2021 were enrolled. Baseline Lp(a) was measured before the operation. CIN was defined as an increase in serum creatinine of more than 25% or 44 μmol within 72 h of contrast administration. The relationship between Lp(a) and CIN risk was analyzed.

**Results:**

A total of 928 T2DM patients were included. CIN developed in 11.1% (103/928) of patients. The Lp(a) level was significantly higher in patients with CIN than in non-CIN patients (311.12 ± 278.66 vs. 254.19 ± 274.56 mg/L, *P* = 0.048). Patients were divided into three groups based on Lp(a) levels: <150 mg/L (*n* = 428), 150 mg/L–300 mg/L (*n* = 266), and ≥300 mg/L (*n* = 234). Each group stratified by increasing Lp(a) concentrations had incrementally greater risks of CIN (7.2% vs. 12% vs. 17.1%, *P* *<* 0.001). Multivariate logistic regression analysis showed that patients with Lp(a) ≥300 mg/L had a 2.41-fold higher risk of CIN than those with Lp(a)< 150 mg/L (OR = 2.41, 95% CI: 1.38–4.21, *P* *=* 0.002). Additionally, for each increase of 1 logarithmic unit in Lp(a), the risk of CIN increased by 1.27 times (OR = 1.27, 95% CI: 1.01–1.64, *P* *=* 0.045).

**Conclusions:**

A higher serum Lp(a) level indicates an increased risk of CIN in T2DM patients undergoing CAG or PCI and can serve as an independent predictor of CIN in this population. This study's findings will aid in the clinical prevention and treatment of contrast agent-induced kidney disease.

## 1 Introduction

Driven by factors such as population aging, obesity, diabetes, and hypertension, the incidence and prevalence of coronary artery disease have been steadily rising (1). Consequently, there has been a parallel increase in the volume and complexity of coronary surgeries. Against this backdrop, the widespread use of iodinated contrast agents in diagnostic and interventional procedures has led to a consequential complication, contrast-induced nephropathy (CIN), which denotes renal injury resulting from exposure to contrast agents. The prevalence of CIN is believed to be influenced by well-established risk factors, with baseline chronic renal insufficiency and diabetes recognized as the most significant contributors (2–6).

Type 2 diabetes mellitus (T2DM) is intricately linked to the development of microvascular and macrovascular complications (7, 8). Given that the kidneys are the primary target organ for microvascular complications of T2DM, it is noteworthy that patients often exhibit concealed kidney damage in the early stages of the disease, rendering renal function fragile and creating a favorable environment for the development of CIN (9, 10). In addition, coronary artery disease is a common macrovascular complication of diabetes, which makes patients more likely to be exposed to contrast-related diagnostic and therapeutic processes. Consequently, numerous studies demonstrate a heightened susceptibility to CIN among patients with T2DM (6, 11–13), with serious consequences, including hospitalization, late mortality, increased mortality, increased risk of complications, and long-term loss of kidney function (14–16). Given the gravity of the situation, there is an evident need for enhanced preventive measures against CIN during contrast agent administration in patients with T2DM. Equally important is the quest for novel predictive markers that can indicate the vulnerability of T2DM patients to developing CIN.

Lipoprotein(a) [Lp(a)] is a distinctive and autonomous serum lipoprotein characterized by a core composition that includes triglycerides, phospholipids, cholesterol, esters, and apolipoprotein B100. Structurally similar to low-density lipoproteins (LDL), Lp(a) is a unique entity within the realm of lipoproteins. With substantial advancements in research in recent years, elevated Lp(a) levels have emerged as an independent risk factor for cardiovascular diseases. Notably, in individuals with diabetes, Lp(a) plays a critical role as a risk factor for coronary atherosclerotic heart disease (17, 18). However, the intricate association between lipids and CIN, particularly with Lp(a), remains unclear. We hypothesized that elevated d. Lp(a) levels independently predict CIN in T2DM patients undergoing CAG or PCI.

## 2 Materials and methods

### 2.1 Study population

**Figure 1:**
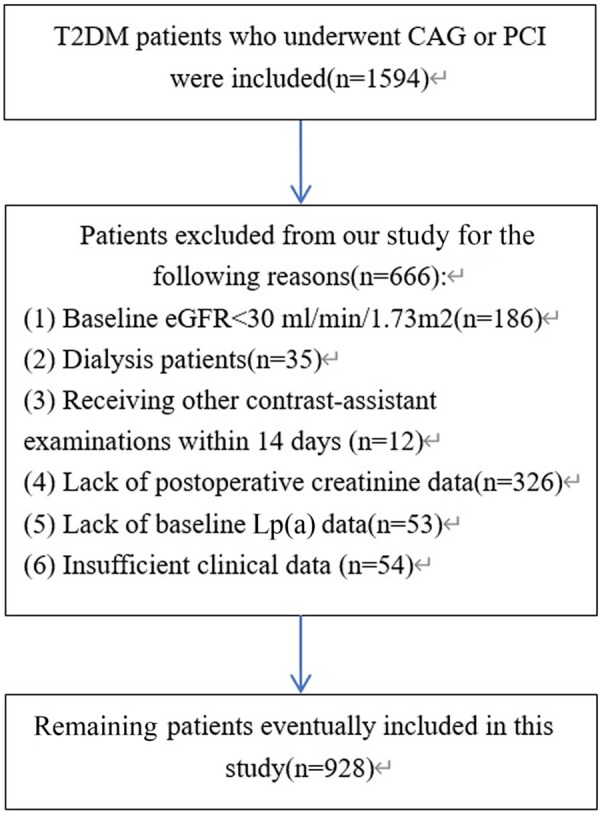
Flow diagram of the study population enrollment and exclusion process.

This retrospective observational study was conducted at the Department of Cardiology, the Third Affiliated Hospital of Sun Yat-sen University. A total of 1,594 consecutive adult patients with T2DM (age ≥18 years) who underwent coronary angiography (CAG) or percutaneous coronary intervention (PCI) were screened for inclusion from January 2019 to December 2021. The diagnosis of T2DM was based on the ADA diabetes guidelines (19). The exclusion criteria were as follows: (1) patients allergic to contrast; (2) baseline eGFR < 30 mL/min/1.73m^2^; (3) patients receiving hemodialysis; (4) patients receiving other contrast-assisted examinations within 14 days; (5) patients with blood pressure lower than 90/60 mmHg or insufficient tissue perfusion; (6) patients with metabolic acidosis, severe infection or injuries, cancer, inflammatory diseases, or autoimmune diseases; (7) patients who had recently taken renal function-impairing medicines or suffered acute kidney injury; and (8) patients with incomplete records of required data. Finally, 928 eligible patients were included in this study (Figure 1). The research protocol was reviewed and approved by the Medical Ethics Committee of the Third Affiliated Hospital of Sun Yat-sen University in accordance with the ethical guidelines of the 1975 Declaration of Helsinki. Written informed consents were obtained from all patients.

### 2.2 Data collection

Baseline data were collected from all participants, including age, gender, body mass index, blood pressure, smoking status, and history of hypertension, coronary artery disease, NYHA classification, and chronic kidney disease. Blood samples were collected after an 8-hour fasting period before catheterization. The following biochemical parameters were measured using a Hitachi 7,180 chemical analyzer (Hitachi High-Tech Corp, Japan): fasting plasma glucose, total cholesterol, triglycerides, high-density lipoprotein cholesterol, low-density lipoprotein cholesterol, lipoprotein (a), baseline serum creatinine, and baseline estimated glomerular filtration rate (eGFR). The eGFR was calculated using the equation developed by the Modification of Diet in Renal Disease Study Group (20). Hemoglobin levels were measured using the cyanmethemoglobin method. Glycosylated hemoglobin A1c levels were measured using high-performance liquid chromatography with the D-10 Hemoglobin Testing System (Bio-Rad Laboratories, Inc., Hercules, CA, USA). Regarding the acquisition of sCr data, the preoperative sCr of the patient was used as the baseline. Medications, including angiotensin receptor neprilysin, angiotensin-converting enzyme inhibitor, sodium-glucose-linked transporter 2 inhibitor, metformin, statin, and insulin, were also recorded. Similarly, the type of operation, contrast medium, and contrast agent doses were fully documented.

### 2.3 Cardiac catheterization and diagnosis of CIN

To protect against intravenous contrast media, all patients received prophylactic intravenous rehydration with 0.9% NaCl solution for 6 h before and after contrast media administration. Unless contraindicated, each patient received adequate doses of aspirin and clopidogrel for antiplatelet therapy before surgery (aspirin: 0.1 g/day for at least 3 days or 0.3 g as a loading dose; clopidogrel: 75 mg/day for at least 4 days or 300 mg as a loading dose) before the operation. CAG or PCI is performed through the radial or femoral artery in accordance with standard clinical practice. The type and dose of the contrast agent were determined by two experienced interventional cardiologists according to the surgical requirements. Sarcosine oxidase enzymatic methods were used to estimate dynamic changes in sCr values at baseline and at 24, 48, and 72 h after coronary catheterization, and accordingly, to obtain the highest Cr value within 72 h after the operation. The clinical outcome was the occurrence of CIN after the administration of contrast media, defined as an increase in sCr concentration of more than 25% or 44 μmol within 72 h of contrast administration, with no evidence of other causes.

### 2.4 Statistical analysis

Statistical analysis was performed using SPSS 26.0 (IBM Corp., Armonk, NY, USA) and R version 4.0.2. Normally distributed data are expressed as mean ± standard deviation and compared using independent-samples *t*-tests. Data indicating poor normality were expressed as interquartile ranges, and rank-sum tests were used for analysis. Continuous variables were analyzed using the Student's *t*-test or Mann–Whitney *U*-test. Categorical variables were tested using the chi-square test or Fisher's exact test when group numbers were small, and the large number assumption for chi-square tests did not apply. Univariate and multivariate logistic regression models were used to assess Lp(a) as an independent predictor of CIN. Unless otherwise noted, a *P*-value <0.05 was considered statistically significant.

## 3 Results

### 3.1 Patient characteristics

The baseline demographic, clinical, and laboratory characteristics of the study population are presented in Table 1. CIN developed in 11.1% (103/928) of patients after CAG or PCI. The Lp(a) level was significantly higher in patients with CIN than in non-CIN patients (311.12 ± 278.66 vs. 254.19 ± 274.56 mg/L, *P* *=* 0.048). In addition, patients with CIN had lower baseline DBP (*P* = 0.041) and eGFR (*P* < 0.001), but higher baseline creatinine (*P* *<* 0.001), TC (*P* = 0.006), and LDL-C (*P* = 0.020) levels than non-CIN participants. Furthermore, the NYHA classification (*P* *=* 0.007), type of contrast medium (*P* = 0.049), and ARNI use (*P* = 0.002) differed between groups.

**Table 1:** Baseline characteristics.

Characteristics	Overall (*n* = 928)	Non-CIN (*n* = 825)	CIN (*n* = 103)	*P* value
Age, y	63.89 ± 10.51	64.00 ± 10.19	63.00 ± 12.80	0.361
Male	642 (69.2)	579 (70.2)	63 (61.2)	0.062
BMI, kg/m^2^	24.50 ± 3.13	24.53 ± 3.11	24.29 ± 3.30	0.464
Hypertension	583 (62.8)	524 (63.5)	59 (57.3)	0.217
Coronary artery disease	800 (86.2)	707 (85.7)	93 (90.3)	0.202
NYHA classification				0.007
Grade I/II	862 (92.9)	773 (93.7)	89 (86.4)	
Grade III/IV	66 (8.6)	52 (6.3)	14 (13.6)	
Chronic kidney disease	387 (26.6)	216 (26.2)	31 (30.1)	0.397
Smoking	420 (45.3)	382 (46.3)	38 (36.9)	0.070
SBP, mmHg	136.52 ± 21.47	136.71 ± 21.51	135.03 ± 21.22	0.455
DBP, mmHg	80.38 ± 12.58	80.68 ± 12.35	78.00 ± 14.12	0.041
HGB, g/L	132.65 ± 18.23	132.79 ± 18.10	131.55 ± 19.29	0.516
Baseline creatinine, µmol/L	72.91 ± 23.79	71.71 ± 23.88	82.50 ± 23.52	<0.001
Baseline eGFR, mL/min/1.73m^2^	80.83 ± 29.76	82.54 ± 33.01	67.11 ± 22.98	<0.001
TC, mmol/L	4.25 ± 1.28	4.21 ± 1.26	4.58 ± 1.39	0.006
TG, mmol/L	1.91 ± 1.66	1.90 ± 1.67	1.94 ± 1.64	0.825
LDL-C, mmol/L	2.80 ± 1.10	2.77 ± 1.06	3.04 ± 1.31	0.020
HDL-C, mmol/L	0.95 ± 0.25	0.95 ± 0.25	0.95 ± 0.26	0.928
Lp(a), mg/L	260.51 ± 275.44	254.19 ± 274.56	311.12 ± 278.66	0.048
FPG, mmol/L	7.33 ± 2.73	7.30 ± 2.71	7.55 ± 2.93	0.378
HbA1c, %	7.30 ± 1.78	7.29 ± 1.75	7.36 ± 2.05	0.717
Type of operation				0.109
CAG only	411 (44.3)	373 (45.2)	38 (36.9)	
CAG + PCI	517 (55.7)	452 (54.8)	65 (63.1)	
Type of contrast medium				0.049
Isotonic	226 (24.4)	209 (25.3)	17 (16.5)	
Low osmolar	702 (75.6)	616 (74.7)	86 (83.5)	
Contrast medium dose, mL	132.50 ± 65.27	131.32 ± 63.74	141.94 ± 76.12	0.119
Medications				
ARNI, *n* (%)	197 (21.2)	187 (22.7)	10 (9.7)	0.002
ACEI/ARB, *n* (%)	276 (29.7)	245 (29.7)	31 (30.1)	0.938
SGLT2i, *n* (%)	113 (12.2)	96 (11.6)	17 (16.5)	0.154
Metformin, *n* (%)	223 (24.0)	197 (23.9)	26 (25.2)	0.760
Insulin, *n* (%)	305 (32.9)	267 (32.4)	38 (36.9)	0.356
Statin, *n* (%)	910 (98.1)	811 (98.3)	99 (96.1)	0.100

CIN, contrast-induced nephropathy; BMI, body mass index; SBP, systolic blood pressure; DBP, diastolic blood pressure; HGB, hemoglobin; TC, total cholesterol; TG, triglycerides; HDL-C, high-density lipoprotein cholesterol; LDL-C, low-density lipoprotein cholesterol; Lp(a), lipoprotein(a); LnLp(a), natural logarithm of Lp(a); FPG, fasting plasma glucose; HbA1c, hemoglobin A1c; CAG, coronary angiography; PCI, percutaneous coronary intervention; ARNI, Angiotensin receptor neprilysin inhibitor; ACEI/ARB, angiotensin-converting enzyme inhibitor/angiotensin receptor blocker; SGLT2i, sodium-glucose cotransporter-2 inhibitor.

Data are presented as mean ± SD, median (25th to 75th percentile), or *n* (%).

### 3.2 Comparison of renal function and CIN occurrence according to Lp(a) levels

According to the reference value range of Lp(a), patients were divided into three groups: Lp(a) < 150 mg/L (*n* = 428), 150 mg/L ≤ Lp(a)< 300 mg/L (*n* = 266), and Lp(a) ≥300 mg/L (*n* = 234). Baseline Cr, baseline eGFR, the highest Cr within 72 h after operation, and occurrence of CIN were counted for these groups (Table 2). No significant differences were observed between the three groups for Baseline Cr, Baseline eGFR, and Highest Cr within 72 h after the operation. However, it appeared that each group stratified by increasing Lp(a) concentrations had incrementally greater risks of CIN (7.2% vs. 12% vs. 17.1%, *P* *<* 0.001).

**Table 2:** Comparison of preoperative and postoperative renal function and CIN occurrence according to Lp(a) levels.

Variable	Lp(a) < 150 (*n* = 428)	150 ≤ Lp(a) < 300 (*n* = 266)	Lp(a) ≥300 (*n* = 234)	*P* value
Baseline Cr, µmol/L	81.24 ± 22.92	81.22 ± 23.76	81.51 ± 25.42	0.988
Baseline eGFR, mL/min/1.73m^2^	68.41 ± 23.85	69.11 ± 25.05	69.25 ± 26.10	0.895
Highest Cr within 72 h after operation, µmol/L	84.71 ± 27.03	86.17 ± 27.69	87.87 ± 33.03	0.399
CIN, *n* (%)	31 (7.2)	32 (12.0)	40 (17.1)	<0.001

### 3.3 Association between Lp(a) and the occurrence of CIN

**Figure 2:**
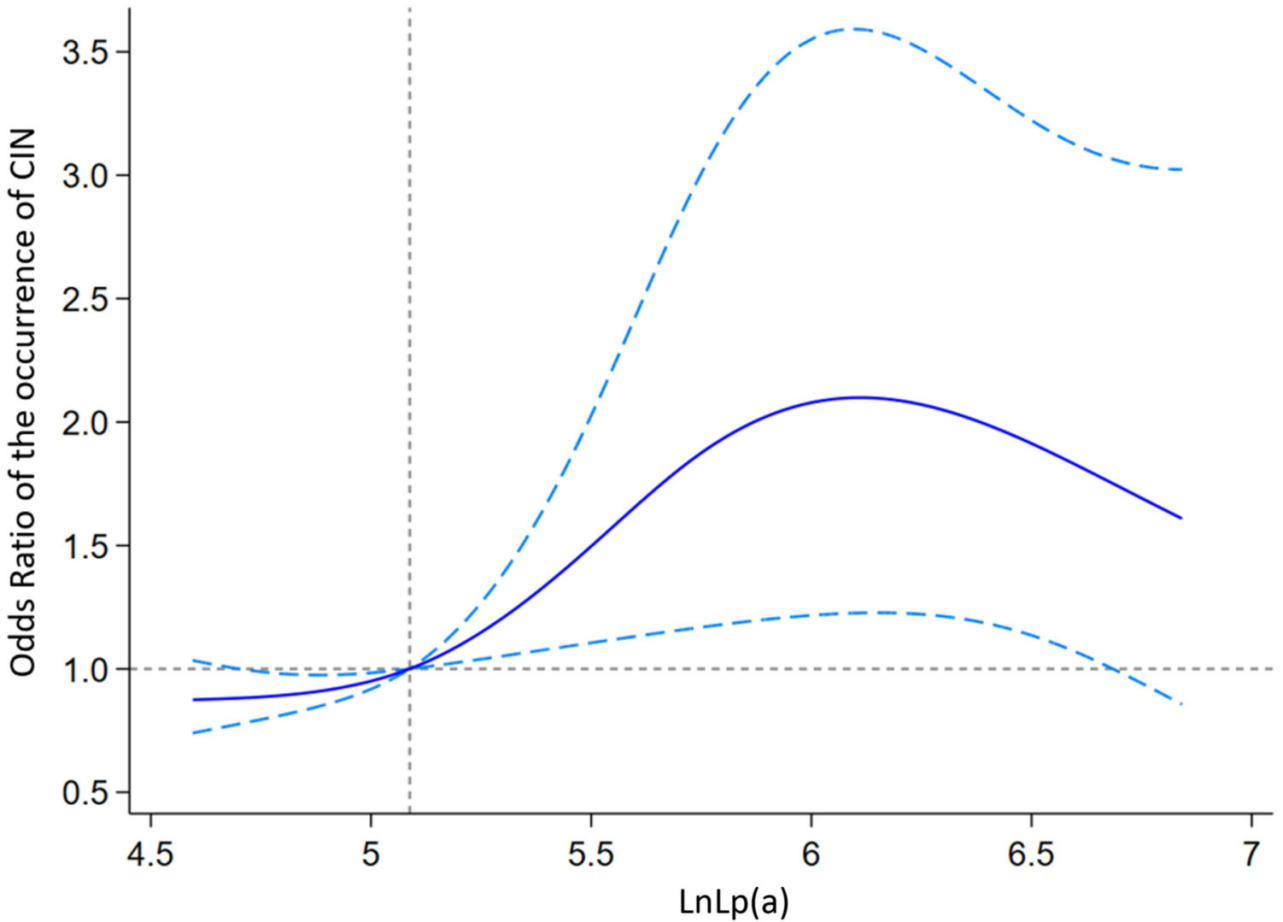
Restricted cubic spline curve illustrating the association between serum Lp**(a)** levels and CIN risk.

Univariate and multivariate logistic regression analyses were performed to analyze the association between Lp(a) levels and CIN occurrence. Categorical Lp(a) was divided into three grades, as described previously, whereas continuous Lp(a) was logarithmically transformed due to its skewed distribution. In the univariate logistic regression analysis (Table 3, Model 1), both 150 mg/L ≤ Lp(a) < 300 mg/L and Lp(a) ≥300 mg/L, compared with Lp(a) <150 mg/L, were potentially associated with the occurrence of CIN (both *P* *<* 0.05). For every increase of 1 logarithmic unit in Lp(a), there was a 1.34-fold increase in the occurrence of CIN (*P* *=* 0.009). In the multivariable logistic regression analysis (Table 3, Models 2 and 3), Lp(a) levels of 150 mg/L ≤ Lp(a) < 300 mg/L showed no significant correlation with the occurrence of CIN (both *P* > 0.05), while Lp(a) ≥300 mg/L was potentially associated with the occurrence of CIN (both *P* *<* 0.05). For each increase of 1 logarithmic unit in Lp(a), the risk of CIN increased by 1.27 times (OR = 1.27, 95% CI: 1.01–1.64, *P* = 0.045) (Table 3, Model 3). These results suggest that elevated Lp(a) levels can serve as independent predictors of CIN in patients with T2DM who underwent CAG or PCI. The restricted cubic spline analysis of the nonlinear trend between Lp(a) and the occurrence of CIN showed that the risk of CIN increased with increasing Lp(a) levels (Figure 2).

**Table 3:** Results of univariate and multivariate logistic regression analysis of Lp(a) and CIN.

Variable	Model 1		Model 2		Model 3	
	OR (95%CI)	*P* value	OR (95%CI)	*P* value	OR (95%CI)	*P* value
Categorical Lp(a)						
Lp(a) < 150	1.00 (reference)	—	1.00 (reference)	—	1.00 (reference)	—
150 ≤ Lp(a) < 300	1.75 (1.04–2.95)	0.035	1.68 (0.99–2.83)	0.052	1.65 (0.95–2.86)	0.075
Lp(a) ≥300	2.64 (1.60–4.35)	<0.001	2.59 (1.56–4.28)	<0.001	2.41 (1.38–4.21)	0.002
Continuous Lp(a)						
per 1 ln-unit increase	1.34 (1.08–1.66)	0.009	1.34 (1.07–1.67)	0.010	1.27 (1.01–1.64)	0.045

Model 1 involved a univariate logistic regression analysis. Model 2 was adjusted for age, gender, and BMI. Model 3 was further adjusted for hypertension, smoking, SBP, DBP, CKD, NYHA classification, baseline eGFR, HGB, TG, HDL-C, LDL-C, HbA1c, FPG, contrast medium type, contrast medium dose, operation type, and drugs. Logarithmic transformation was applied to continuous Lp(a) in the table due to its skewed distribution.

### 3.4 Subgroup analysis

Next, we conducted subgroup analyses using Lp(a) as a categorical or continuous variable to investigate the relationship between Lp(a) and the occurrence of CIN within different subgroups defined by gender, age, hypertension, HbA1c, baseline eGFR, operation, and contrast medium.

**Figure 3:**
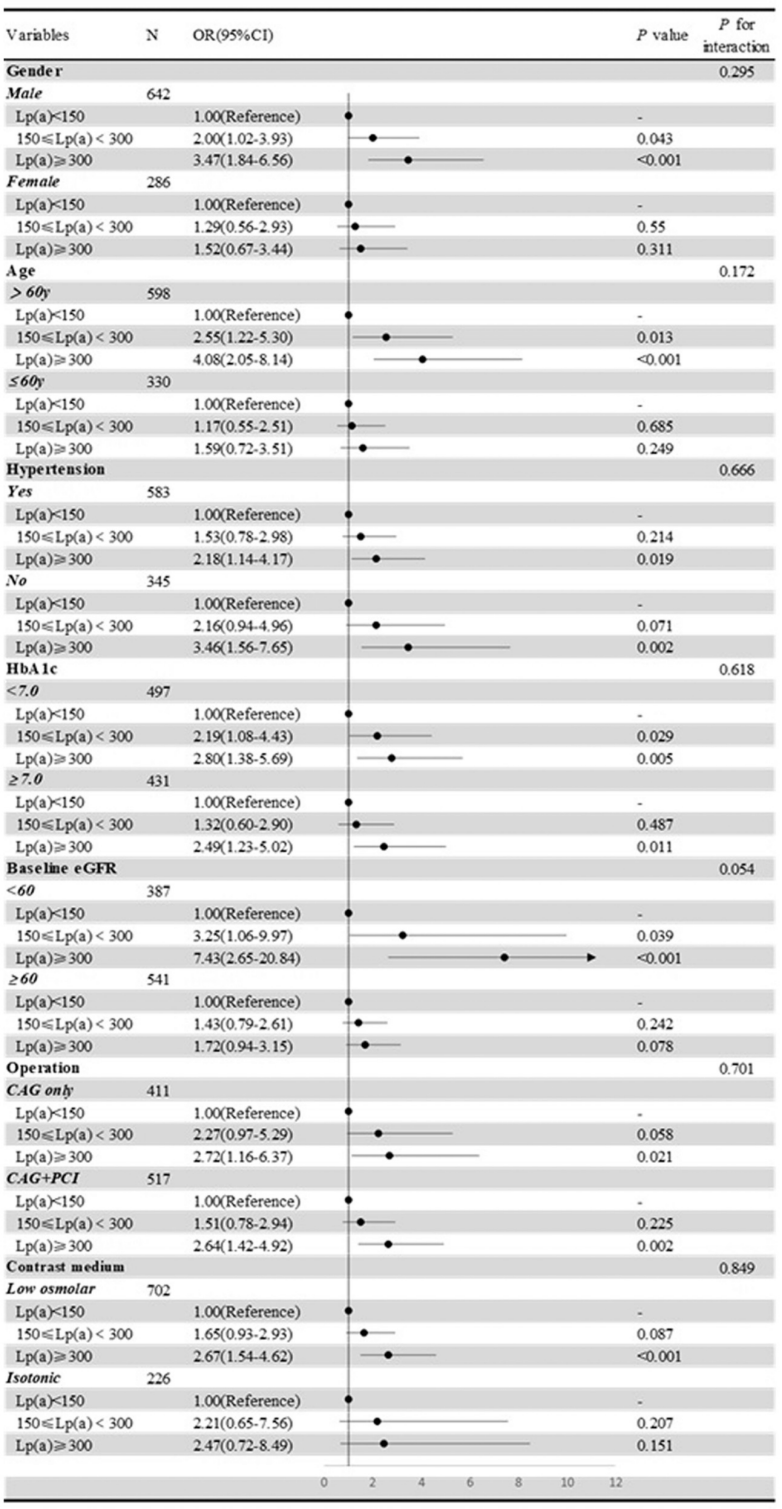
Subgroup analysis of the association between Lp**(a)** and CIN risk, treated as a categorical variable.

In the subgroup analysis using Lp(a) as a categorical variable (Figure 3), 150 mg/L ≤ Lp(a) < 300 mg/L was associated with an increased occurrence of CIN in subgroups of males (*P* = 0.043), those aged >60 years (*P* = 0.013), those with HbA1c <7.0 (*P* = 0.029), and those with baseline eGFR <60 mL/min/1.73m^2^ (*P* *=* 0.039). In addition, Lp(a) ≥300 mg/L was associated with an increased occurrence of CIN in subgroups of males (*P* *<* 0.001), those aged >60 years (*P* *<* 0.001), those with a baseline eGFR < 60 mL/min/1.73 m^2^ (*P* *<* 0.001), and those with low osmolar contrast medium (*P* *<* 0.001). In all subgroups of hypertension, HbA1c level, and operation type, Lp (a) ≥300 mg/L was associated with an increased incidence of CIN (all *P* *<* 0.05).

**Figure 4:**
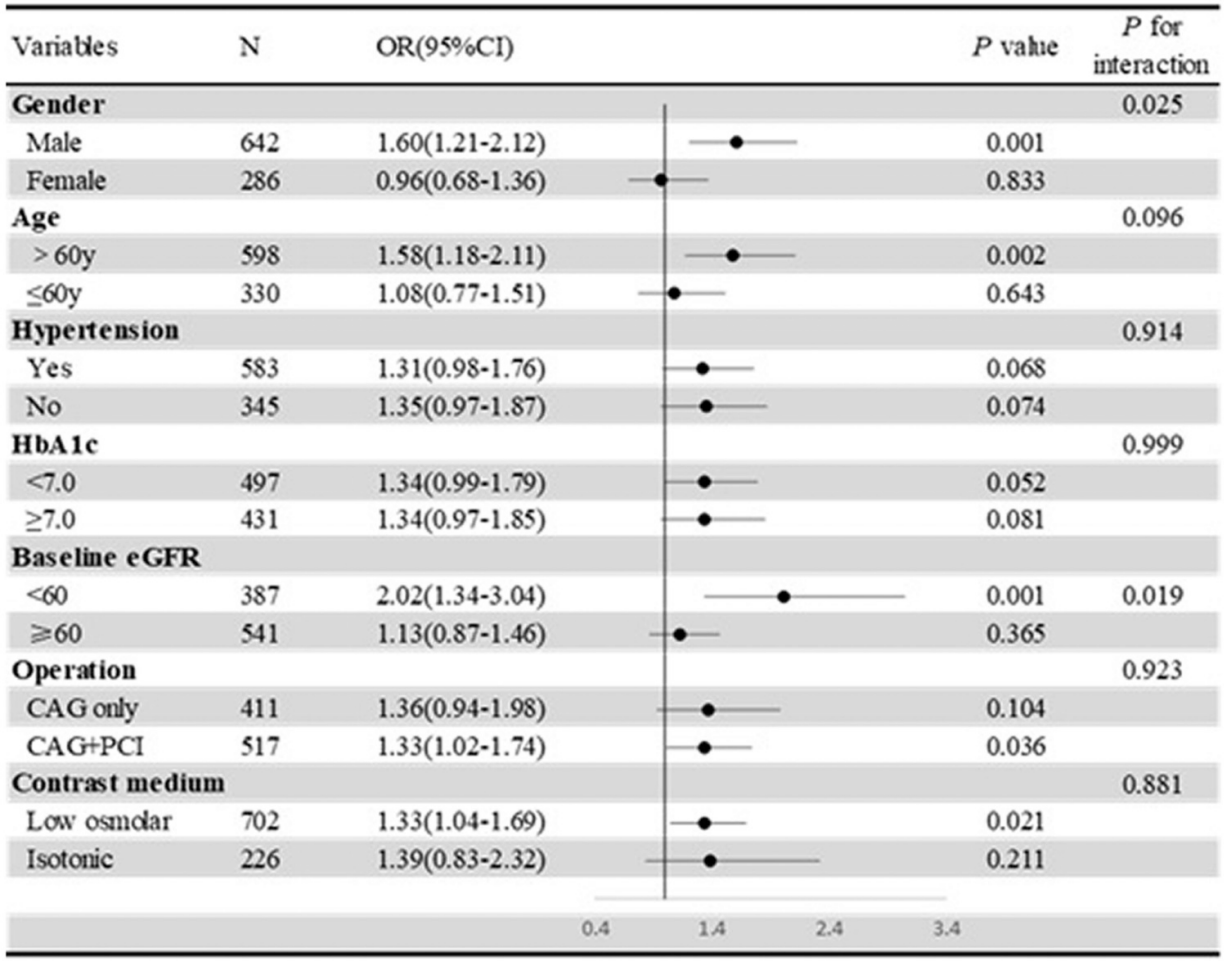
Subgroup analysis of the association between Lp**(a)** and CIN risk, treated as a continuous variable.

In the subgroup analysis using Lp(a) as a continuous variable (Figure 4), Lp (a) was associated with the occurrence of CIN in the subgroups of males (*P* = 0.001), those aged >60 years (*P* = 0.002), those with a baseline eGFR < 60 mL/min/1.73m^2^ (*P* = 0.001), those who underwent CAG + PCI procedures (*P* = 0.036), and those who received low osmolar contrast medium (*P* = 0.021).

## 4 Discussion

In this study, we investigated for the first time the association between serum Lp(a) levels and the occurrence of CIN. We demonstrated that elevated Lp(a) levels are independent predictors of CIN development in T2DM patients undergoing CAG or PCI procedures. Serum Lp(a), a routinely measured, fast, and simple blood lipid marker, has significant value in helping clinicians balance the benefits and risks of contrast agent administration in patients with T2DM before risk scoring and stratification, thereby promoting the prevention of CIN.

T2DM is intricately intertwined with the occurrence of coronary microvascular complications (21, 22). The management of these complications often involves procedures such as CAG or PCI, exposing patients to a heightened risk of contrast agent exposure. Currently, the status of T2DM as a major risk factor for CIN has been extensively documented in the literature (3–6, 14, 23, 24). Both diabetes and contrast agent administration significantly impact renal physiology, including renal hemodynamics, tubular transport activity, oxygen consumption, as well as medullary hypoxia and increased ROS production (10, 14, 25, 26). These factors collectively disrupt the protective mechanisms, thereby increasing CIN susceptibility. Considering these findings, it is imperative to incorporate expanded predictive factors into the preoperative assessment of CIN risk in patients with T2DM. Such an approach is crucial for enhancing risk stratification and optimizing clinical decision-making.

The current diagnosis of CIN relies on serum creatinine (sCr) levels, which have several inherent limitations as markers of renal function. These limitations include, but are not limited to, the following: 1. sCr is a late marker of renal injury, as renal function impairment occurs prior to an increase in sCr levels (27, 28). 2. sCr levels are influenced by non-renal factors such as age, gender, muscle mass, hydration status, and therapeutic medications (29). 3. However, sCr cannot accurately depict renal function until it reaches steady state. Several novel, highly sensitive biomarkers have been identified as predictive markers for CIN. These emerging predictive factors include, but are not limited to, plasma volume status (PVS) (30), serum uric acid/albumin ratio (31), and antithrombin III (32). We propose that Lp(a) plays a significant predictive role in CIN in patients with T2DM undergoing CAG or PCI. Our findings highlight the importance of Lp(a) as a potential biomarker for risk stratification and prognosis in this specific patient population, offering valuable insights into personalized approaches for CIN prevention and management.

Research on plasma lipoproteins as early detection biomarkers or predictive factors for acute kidney injury (AKI) caused by various etiologies has focused on neutrophil gelatinase-associated lipocalin (NGAL) in plasma (33–35). In comparison, Lp(a) has emerged as a routine plasma biochemical marker and shows considerable sensitivity and specificity for predicting CIN in the population under investigation. The possible underlying mechanisms include: 1. Lp(a) can induce an inflammatory response, stimulating the production of inflammatory cells and cytokines (36). This further contributes to inflammatory injury and cellular apoptosis in the renal tissue; 2. Lp(a) facilitates platelet activation and aggregation, leading to the formation of micro-clots (37). These clots can obstruct the renal microvasculature, leading to renal ischemia and hypoxia and thereby promoting the onset of CIN; 3. Lp(a) can induce oxidative stress, leading to increased production of intracellular free radicals (38). These radicals directly damage renal cells and trigger inflammatory responses in the kidneys. Additionally, Lp(a) can inhibit antioxidant enzyme activity, further exacerbating oxidative stress; 4. Lp(a) can impair vascular endothelial function, leading to injury and increased vessel wall permeability (39). This may result in the leakage of plasma components and inflammatory mediators into the glomeruli and renal tubules, thereby triggering CIN.

As research progresses, it is possible that Lp(a) will be clinically incorporated into preoperative risk assessment systems for CIN.

## 5 Study limitations

This study has several limitations. First, This is a single-center observational study, which limits the applicability of causal inferences. And further trials in more diverse populations are required to validate these findings. Second, this study focused specifically on patients with diabetes; variations in glycemic control and disease duration may have influenced the observed outcomes. Additionally, variations were observed in the specific preventive measures for CIN implemented among different patients. Finally, as the pathophysiological link between Lp(a) and CIN remains unclear, further research is necessary to elucidate the potential molecular mechanisms underlying this association. Third, this study focused on diabetic patients because diabetic patients are at a high risk of developing contrast-induced nephropathy and suffer greatly from it. The next step will be to conduct further research to explore the correlation of Lp(a) with the development of contrast-induced nephropathy in non-diabetic patients, in order to gain a more comprehensive understanding of Lp(a) as a risk factor for contrast-induced nephropathy. Forth, while there were more male participants in the study, there were also some female patients, so it's possible that the findings may be more representative of males. Sixth, the implementation of preventive measures (hydration, medication) in this study was not uniform and may have resulted in some variations.

## 6 Conclusion

In conclusion, higher serum Lp(a) levels are associated with an increased risk of CIN in T2DM patients undergoing CAG or PCI procedures. Serum Lp(a) serves as an independent predictor of CIN in this population, providing a valuable addition to existing clinical risk-scoring models. Preoperative measurement of Lp(a), a routine and accessible biomarker, can assist clinicians in performing more accurate risk stratification and balancing the benefits and risks of contrast exposure, ultimately facilitating the prevention of CIN in patients with T2DM.

## Data Availability

The raw data supporting the conclusions of this article will be made available by the authors, without undue reservation.
